# Influence of Cold Storage on Pear Physico-Chemical Traits and Antioxidant Systems in Relation to Superficial Scald Development

**DOI:** 10.3390/foods9091175

**Published:** 2020-08-25

**Authors:** Giuseppina Caracciolo, Anna Magri, Milena Petriccione, Maria Luigia Maltoni, Gianluca Baruzzi

**Affiliations:** 1 Consiglio per la Ricerca in Agricoltura e l’Analisi dell’Economia agraria (CREA), Centro di ricerca Olivicoltura, Frutticoltura e Agrumicoltura, 47121 Forlì, Italy; marialuigia.maltoni@crea.gov.it (M.L.M.); gianluca.baruzzi@crea.gov.it (G.B.); 2 Consiglio per la Ricerca in Agricoltura e l’Analisi dell’Economia agraria (CREA), Centro di ricerca Olivicoltura, Frutticoltura e Agrumicoltura, 81100 Caserta, Italy; annamagri93@gmail.com (A.M.); milena.petriccione@crea.gov.it (M.P.)

**Keywords:** pear cultivars and advanced selections, superficial scald, oxidative stress, physico-chemical traits

## Abstract

Superficial scald is the most common physiological disorder in apples and pears and causes huge economic losses worldwide. The aim of this study is to determine the different scald susceptibilities of seven pear cultivars/selections during five months of cold storage (CS). Four advanced pear selections and three commercial cultivars were harvested from an orchard located in Emilia-Romagna region, and cold stored at −1 °C and 85% relative humidity (RH).After 90, 120, and 150 days, fruits of each cultivar and selection were removed for ripening for 4 d, after which scald damage, physico-chemical and nutraceutical traits, and enzymatic antioxidant systems were evaluated on peel and pulp. ‘Abbé Fétel’, ‘Falstaff’, and ‘CREA 171’ did not showed superficial scald symptoms after 90 days, while ‘Doyenne du Comice’ and ‘CREA 264’ showed the highest susceptibility. After 90 days, CS ‘Falstaff’ and ‘CREA 179’ showed the highest total polyphenol content (TPH) in peel, followed by ‘Doyenne du Comice’ and ‘AbbéFétel’; lowest TPH was detected in ‘CREA 264’. After 120 and 150 days of CS, ‘Abbé Fétel’ and ‘CREA 171’ showed the highest peel TPH. ‘CREA 264’ and ‘CREA 125’ reached the lowest values of TPH during the three CS time periods. Superoxide dismutase and catalase activities were higher in the peel of scald-resistant than that in scald-susceptible pear cultivars/advanced selections. Superficial scald induced an increase in polyphenoloxidase, and guaiacol peroxidase activities involved in fruit-browning during CS. Furthermore, we observed an increase in lipoxygenase activity and consequent membrane damage in both the peel and flesh of the fruit. This study indicates that pear cultivars/advanced selections have different superficial scald susceptibilities that enable them to induce the activity of several antioxidant enzymes, following CS.

## 1. Introduction

Superficial scald is the most common physiological disorder of pome fruits such as apples and pears and causes huge economic losses worldwide [1]. The susceptibility of a fruit to scald depends on pre-harvest factors, low temperatures during fruit storage, and genetic traits of cultivars. The symptoms are characterized by the onset of irregular brown or black patches on the surface layers of hypodermal cortical tissue cells, which renders the fruit unmarketable [2,3].

Complex networks and multiple key factors are involved in fruit superficial scald development, such as ethylene metabolism, α-farnesene content, hydroperoxides or conjugated trienols (CTols), reactive oxygen species (ROS), and programmed cell death (PCD); however, other physiological or biochemical processes that determine scald susceptibility are not equivalent in apples and pears [2,4,5,6]. No positive correlation has been found between ethylene production and scald incidence in pears, as demonstrated in the ‘Beurréd’Anjou’ pear that contains a low amount of ethylene but high scald susceptibility [7]. Furthermore, several studies have demonstrated that α-farnesene biosynthesis could be not ethylene-dependent but influenced also by pre- and post-harvest factors in different pear cultivars [4,7,8,9].

ROS and antioxidant systems are likely to be involved, at least in part, in scald development [2,10]. In fruits, the antioxidant system includes an enzymatic and a non-enzymatic component that play an important role in preventing the oxidation of α-farnesene, thus modulating fruit superficial scald development [2,10].

Pre-harvest factors such as early timing of ripening stage and picking time contribute to a greater incidence of scald development due to reduced activity of the antioxidant system associated with faster accumulation of oxidation products in less ripe fruit [8]. Furthermore, pre-harvest sunlight exposure delays the onset of scald in the ‘d’Anjou’ pear, by improving antioxidant contents and antioxidant enzyme activities in blushed peel at harvest and during storage [11]. Post-harvest factors such as chilling stress cause an increase in reactive oxygen species (ROS) content that contributes to α-farnesene oxidation, aggravating symptoms in fruits which have already been compromised by oxidative stress [6]. This post-harvest disorder should not appear during storage if fruit maintains a high antioxidant content that prevents or limits α-farnesene oxidation [12,13].

This oxidative phenomenon occurs during post-harvest in fruits due to the production and removal of reactive oxygen species (ROS), such as hydroxyl radicals H_2_O_2_ and O_2_^−^_,_ from the tissues [14]. Non-enzymatic antioxidants can counteract oxidation-linked damages responsible for superficial scald through both stimulation of antioxidant enzymes responses that the biosynthesis of less-oxidisedphenolics that are involved in protective redox-linked pathways [15]. The failure or reduction in capability of the antioxidant system, which includes enzymes ascorbate peroxidase (APX), catalase (CAT), peroxidase (GPX), and superoxide dismutase (SOD), could cause an increase in ROS levels, with inducing oxidative damage to the membrane and subsequently fruit superficial scald development [11,16,17].

Few recent studies have been carried out to elucidate the relationship among antioxidant enzyme activity and scald development in pears. In the blushed peel of ‘d’Anjou’ pears, SOD and APX activities were positively correlated with scald susceptibility, while CAT activity was not related to the surface area of scald [11]. Both ‘Wujiuxiang’ and Yali pears registered a low incidence of scald, associated with an increase in APX, SOD, and CAT activity following 1-methylcyclopropene (1-MCP) post-harvest treatment [18] and 1-MCP combined with modified atmosphere packaging (MAP) [19]. Furthermore, a higher scald susceptibility was related to polyphenoloxidase (PPO) and lipoxygenase (LOX) activities in Japanese [16,17] and Yali pears [19], while POD activity had no correlation to the incidence of superficial scald [16].

In Italy, the European pear (*Pyrus communis* L.) is an important crop which produces approximately 717,000 tons of produce per year, representing 23% of the total European production and 3% of the worldwide production [20]. ‘Abate Fétel’ is a winter cultivar and is the most important pear cultivar in Italy, accounting for about 41% of the total national production. Several studies showed ‘Abbé Fétel’ has a low susceptibility to superficial scald [21,22,23,24]. Its fruits are appreciated by consumers for its aroma, texture, and balanced sweet and sour taste, which are maintained during cold storage (CS) [25]. ‘Doyenne du Comice’ is another winter pear cultivar with a unique eating quality, which is also susceptible to scald [26].

An Italian pear breeding program has been carried out by CREA-Research Centre for Olive, Fruit and Citrus Crops in Forlì (Italy), through which several cultivars have been developed and released, such as ‘Falstaff’, a winter cultivar with a shape similar to ‘Abate Fétel’ but with red skin, juicy flesh, and a high sugar level combined with an acidic taste. One of the objectives of the program is to identify new cultivars with low susceptibility to superficial scald under refrigeration conditions normally used to extend storability. Furthermore, advanced selections are still undergoing evaluation; where some advanced selections have shown valid agronomic and qualitative performances, such as ‘CREA 125’, obtained from a cross of ‘Conference’ and ‘PirosWilmos’, which ripens in mid-August. Its fruits are medium-sized, with an orange-red overcolor on about 70% of the surface. ‘CREA 171’ obtained from a cross of ‘Super Elliott’ × ‘Max Red Bartlett’, and is harvested in mid-late September. Fruits are medium-to-large sized, with good storability. During CS their dark color lightens slightly, making the fruit more attractive; this fruit seem to have low susceptibility to scald. ‘CREA 179’ is an autumn-winter selection (harvested the last week of August) obtained from a ‘Cascade’ × ‘PirosWilmos’ cross. It has a red-skin and medium-sized fruits. ‘CREA 264’ is a selection obtained from a ‘Harrow Sweet × Turandot’ cross, the harvesting period is mid-August, the tree is very easy to manage and produces large red-blushed fruits, which seem to be susceptible to scald.

A preliminary study by our research group has demonstrated different scald susceptibilities in pear cultivars such as ‘Abbé Fétel’ and ‘Doyenne du Comice’, and selections such as CREA 171 and CREA 264 [27], but further investigation is needed to elucidate scald development in other pear cultivars and selections during CS.

The aim of this study is to analyze the physico-chemical, qualitative, and enzymatic traits of four pear selections and three commercial pear cultivars to determine their different scald susceptibilities, and to elucidate the role of antioxidant systems and oxidative damages in modulating susceptibility/resistance to superficial scald development in these pear samples.

## 2. Materials and Methods

### 2.1. Fruit Material and Storage

During the harvest season (August–September) of 2018, in an orchard located in the Emilia-Romagna region (Campogalliano, Italy), four advanced pear selections obtained from the Breeding Program of CREA, which were ‘CREA 264’, ‘CREA 125’, ‘CREA 171’, and ‘CREA 179’, and three commercial cultivars ‘Abbé Fétel’, ‘Doyenne du Comice’, and ‘Falstaff’, were picked at commercial maturity (flesh firmness of 5–6 kg 0.5 cm^−2^ depending on the cultivar/selection), as indicated in Table 1. The cultivars and selections were cultivated following the traditional cultivation technique of the area; using a spindle canopy system with tree spacing of 3.7 m × 1.5 m. The orchard was irrigated, with inter-row grass, processing/weeding along the row, and an anti-hail net covering the trees.

At harvest, 20 fruits of each cultivar and selection were analyzed for physico-chemical and nutraceutical traits, and for scald susceptibility by evaluating the enzymatic antioxidant system.

Representative fruit samples of marketable ripe fruits of the seven cultivars/selections (sixty fruits for each accession) were placed in cold storage at −1 °C and 85% RH.

After 90, 120, and 150 days at −1 °C ± 0.5 °C, 20 fruits of each cultivar and selection were removed and left to ripen for 4 d, after which the scald damage was evaluated, moreover, peel and pulp pears were analyzed for physico-chemical and nutraceutical traits, and for scald susceptibility by evaluating the enzymatic antioxidant system.

#### 2.1.1. Scald Evaluation

Scald index measurement was expressed as a percentage of the fruit surface area affected, where no scald = 0, slight < 25% = 1, moderate 25–50% = 2, and severe > 50% = 3, and was normalized to 100 by multiplying values by 33.3 (100:3) [28].

#### 2.1.2. Physico-Chemical Traits

Peel color, soluble solids content (SS), titratable acidity (TA), and pH were evaluated as quality traits for each cultivar and selection, at each evaluation date after CS and after 3 d at 20 °C. Twenty fruits for each accession and timing were analyzed.

A Minolta chroma meter CR-200 with an 8 mm window was used to provide the three-color coordinates: L*, a*, and b*referred to peel color. The instrument was calibrated with standard white (Y = 93.96; x= 0.3138; y = 0.3214). Color changes from green to yellow were indicated by calculating the Hue angle (H°), as tan^−1^ b*/a* in degrees, and chroma, representing color saturation or vividness, was calculated as (a*^2^ + b*^2^)^1/2^ [29]. After recording the above-mentioned measure, fruits were squeezed by a manual fruit squeezer, and four replicates for each thesis (accession × date) were considered. The collected juices were used to evaluate the SS content, measured by an automatic refractometer (AtagoTM Smart-1, Tokyo, Japan) and expressed as °Brix. TA values were determined by titrating juice samples, up to pH 7.0, with 0.1 M NaOH solution using an automatic titration system (702 SM Titrino-Metrohm, Milan, Italy), that was also used as pH meter. TA was expressed as g malic acid L^−1^.

#### 2.1.3. Total Phenolic Content (TPH)

Peel and pulp for each pear cultivar/advanced selection (4 fruits) were freeze-dried and powdered using an analytical IKA^®^ A11 (Teramo, Italy) basic mill. The extraction was carried out following the method described by Proteggente et al. [30]. For each biological replicate, three technical replicates were prepared.

TPH in pear extract was evaluated using Folin–Ciocalteu reagent, according to the method of Slinkard and Singleton [31]. Gallic acid was used as the standard and a calibration curve was calculated using standard solutions ranging from 10 to 200 mg of gallic acid per liter. A spectrophotometer, GenesysTM 10 (Thermo, Waltham, MA, USA), was used as a detector and the assay was realized at 750 nm. The results were expressed as milligrams of gallic acid equivalent (GAE) per g of dry weight (DW).

#### 2.1.4. Total Antioxidant Activity (TAA)

Total antioxidant activity (TAA) was determined according to the method of Re et al. [32], based on the oxidation of ABTS by potassium persulfate to form a radical cation ABTS●+. Trolox^®^ was used as standard. The calibration curve was calculated on the inhibition percentage of standard solutions ranging from 1.8 to 18 µM. A spectrophotometer, GenesysTM 10 (Thermo), was used as a detector and the detection was realized using a wavelength at 734 nm. The results were expressed as Trolox^®^equivalent (TE) per g of dry weight (DW).

#### 2.1.5. Enzyme Extraction and Activity Assays

Extract of total soluble proteins were obtained blending frozen pears (peel and flesh) powder in a solution containing 0.1 M of potassium phosphate (pH 7), 1 mM of sodium-ethylenediaminetetraacetic acid (EDTA pH 7), 6.25 mM of polyethylene glycol (PEG), 5% (*w*/*v*),polyvinylpolypyrrolidone (PVPP), and 5 mM of ascorbic acid (only for APX enzyme assay). After centrifugation at 14,000× *g* for 20 min at 4 °C, of the crude extract obtained, were measured soluble protein content through the Bradford assay [33], catalase (CAT), superoxide dismutase (SOD), ascorbate peroxidase (APX) and guaiacol peroxidase (GPX) activity.

Catalase (EC 1.11.1.6) activity was assayed according to the method described by Magri et al. [34] with slight modifications. The mixture for reaction containing potassium phosphate buffer (100 mM; pH 7), H_2_O_2_ (20 mM) and crude enzyme extract. The decrease in absorbance at 240 nm was registered after adding of H_2_O_2_, that promoting reaction start. CAT activity was expressed as μmol of H_2_O_2_ per g^−1^ FW.

Superoxide dismutase (EC 1.15.1.1) activity was evaluated in according to Petriccione et al. [27]. The mixture used for reaction included potassium phosphate buffer (50mM, pH 7.8), sodium EDTA (0.1 mM, pH 7), methionine (13mM), nitro blue tetrazolium chloride (NBT, 75 mM), riboflavin (75 µM), and crude enzyme extract. The absorbance was registered at 560nm, afterwards 10 min of incubation at room temperature under continuous light. SOD activity was expressed as U g^−1^ of FW. One SOD unit corresponds to the amount of enzyme that occurs to inhibits the rate of NBT reduction by 50%, under the experimental conditions.

Ascorbate peroxidase (EC 1.11.1.11) activity was determined in according to the method described by Goffi et al. [35]. The reaction mixture contained potassium phosphate buffer (0.1 M, pH 7), sodium EDTA (0.66 mM, pH 7), ascorbic acid (0.33 mM), H_2_O_2_ (0.35 mM), and crude enzyme extract. Ascorbic acid oxidation was detected spectrophotometrically at 290 nm and APX activity was expressed as μmol of ascorbate per g^−1^ FW.

Guaiacol peroxidase (EC 1.11.1.7) activity was obtained according to the method described by Modesti et al. [36]. The reaction was carried out in the presence of potassium phosphate buffer (0.1M; pH 7), sodium-EDTA (0.15 mM; pH 7.0), H_2_O_2_ (6.66mM), guaiacol (8 mM) and crude enzyme extract. Guaiacol peroxidase activity was registered spectrophotometrically at 470 nm, monitoring the tetraguaiacol formation. GPX activity was expressed as μmol per g^−1^ FW.

Polyphenoloxidase activity (EC.1.10.3.1; PPO) was carried out homogenizing peel and flesh of pears in a buffer containing sodium phosphate buffer (100 mM, pH 6.4) and 0.05 g of PVPP. The activity was determined thought the method described by Adiletta et al. [37], with slight modifications, using as substrate reaction the catechol (500 mM) dissolved in sodium phosphate buffer (100 mM, pH 6.4). PPO activity was expressed as μmol per g^−1^ FW.

Lipoxygenase activity (LOX) was determined as described by Petriccione et al. [38] with slight modifications. Frozen peel and flesh powder were homogenized in potassium phosphate buffer (0.05 M, pH 7.8), sodium-EDTA (0.001 M, pH 7), and 2% PVPP. After centrifugation at 12,500× *g* for 10 min at 4 °C, the supernatant was used to assay. The mixture of reaction was composed of sodium phosphate buffer (0.1 M, pH 6), linoleic acid sodium salt (0.005 M) and crude enzyme extract. LOX activity was registered spectrophotometrically like the increase in absorbance at 234 nm due to hydroperoxides formation. LOX was expressed as nmol of hydroperoxides per g^−1^ FW.

### 2.2. Statistical Analysis

Data are expressed as the mean ±standard deviation (SD). Difference between several pear cultivars and selections during CS were evaluated by one-way ANOVA and the Duncan (LSD) test for mean comparisons were used. Statistical comparisons were made within each timing. Differences at *p* < 0.05 were considered significant and are indicated with different letters. A principal component analysis (PCA) was used to describe the relationship between the physico-chemical, nutraceutical and enzymatic traits and to identify the principal components that accounted for the majority of the variation within the dataset. Before PCA analysis, data have been normalized. All analyses were performed using the SPSS software package, version 20.0 (SPSS Inc., Chicago, IL, USA).

## 3. Results and Discussion

### 3.1. Physico-Chemical Traits of Different Cultivar/Selection Pears During CS

Pear cultivars and selections were picked in mid-August (‘CREA 125’, ’CREA 179’, and ‘CREA 264’) and September (‘AbbéFétel’, ‘Falstaff’, ‘Doyenne du Comice’, and ‘CREA 171’) (Table 1). At harvest, ‘CREA 171’ (15.50 ± 0.15) showed the highest TSS content and was statistically different from other cultivars and selections. ‘Doyenne du Comice’ (11.55 ± 0.10) showed the lowest TSS content. After the shelf-life periods that followed cold storage, TSS increased in all cultivars/selections. After 90 days of CS, ‘CREA 171’ (16.56 ± 0.77 °Brix) and ‘Abbé Fétel’ (15.62 ± 0.91 °Brix) showed the highest TSS content, with a weak statistical difference, followed by ‘Falstaff’ (14.97 ± 0.39 °Brix) and ‘CREA 264’ (14.90 ± 0.56 °Brix), while ‘CREA 125’ showed the lowest TSS content. After 120 days, ‘CREA 171’ (17.15 ± 1.07 °Brix) and ‘CREA 125’ (13.46 ± 0.40 °Brix) were confirmed as having the highest and lowest TSS content, respectively, and were statistically different from all other tested cultivars/selections. After 150 days of CS, ‘CREA 171’ (17.77 ± 0.73 °Brix) reached the highest TSS content followed by ‘Abbé Fétel’ (17.15 ± 1.07 °Brix) and ‘Falstaff’ (17.07 ± 1.65 °Brix), although these differences were not statistically significant. TSS increased in response to the ripening process during CS. This change increases the perceived fruit sweetness, thereby strongly influencing the fruit’s taste [39]. Sugar composition differs between pear cultivars and the increase in TSS could be due to alterations in the cell wall structure and the breakdown of complex carbohydrates into simple sugars [40].

Regarding pulp acidity during CS, TA decreased, and pH increased for all cultivars/selections. At harvest ‘CREA 171’ (3.91 ± 0.05) showed the highest TA, followed by ‘CREA 179’ (3.28 ± 0.23), while ‘Abbé Fétel’ (1.84 ± 0.09) showed the lowest malic acid content. After 90 days ‘CREA 171’ (3.65 ± 0.48 g malic acid/L) showed the highest TA and was statistically different from other cultivars and selections. ‘Abbé Fétel’ (1.58 ± 0.44 g malic acid/L) and ‘Falstaff’ (1.91 ± 0.29 g malic acid/L) showed the lowest malic acid content. After 30 d, ‘CREA 171’ (3.25 ± 0.63 g malic acid/L) displayed the highest sourness, followed by ‘CREA 179’ (3.03 ± 0.17 g malic acid/L), without statistical significance. After 150 days, ‘CREA 171’ (1.15 ± 0.15 g malic acid/L) were subjected to a significant decrease in sourness (−2.10 g malic acid/L in one month), reaching the lowest value of detached acidity (1.15± 0.15 g malic acid/L), while ‘CREA 179’ (2.55 ± 0.13 g malic acid/L) showed the highest sourness value.

TA evaluates the organic acid content of fleshy fruits and is an important trait involved in fruit organoleptic quality. The main organic acids found in most ripe fruits are malic and citric acid [41]. In all pear cultivars, malic acid is the most widely found organic acid, although the composition of organic acids varies more than that of sugars [42].

During CS, the incidence of superficial scald was significantly different among different pear cultivars and selections. ‘Abbé Fétel’, ‘Falstaff’, and ‘CREA 171’ did not show superficial scald symptoms after 90 days, while ‘Doyenne du Comice’ and ‘CREA 264’ showed the highest susceptibility. The first symptoms of superficial scald appeared after 120 days in ‘AbbéFétel’, ‘Falstaff’, and ‘CREA 171’, like in the fruit of ‘CREA 125’, although at significantly lower rates than that in ‘Doyenne du Comice’, ‘CREA 179’, and ‘CREA 264’. After 150 days CS symptoms of superficial scald reached the maximum value in ‘CREA 264’ and ‘Doyenne du Comice’ fruits, and they were not evaluated for physico-chemical traits. ‘Abbé Fétel’, ‘CREA 179’, ‘CREA 171’, and ‘Falstaff’ showed the lowest susceptibility to superficial scald (Table 2). These data agreed with that of our previous study [27] and confirm that scald susceptibility in pears is cultivar dependent. Larrigaudière and co-workers [7] proved that ‘Beurréd’Anjou’ fruits were more susceptible than those of ‘Packham Triumph’ and that the standard parameters used to evaluate fruit ripeness at harvest cannot predict the differences in scald susceptibility between two pear cultivars, as previously described [8].

Changes in skin color were affected by pear cultivar/selection and post-harvest storage conditions [24].

**Figure 1:**
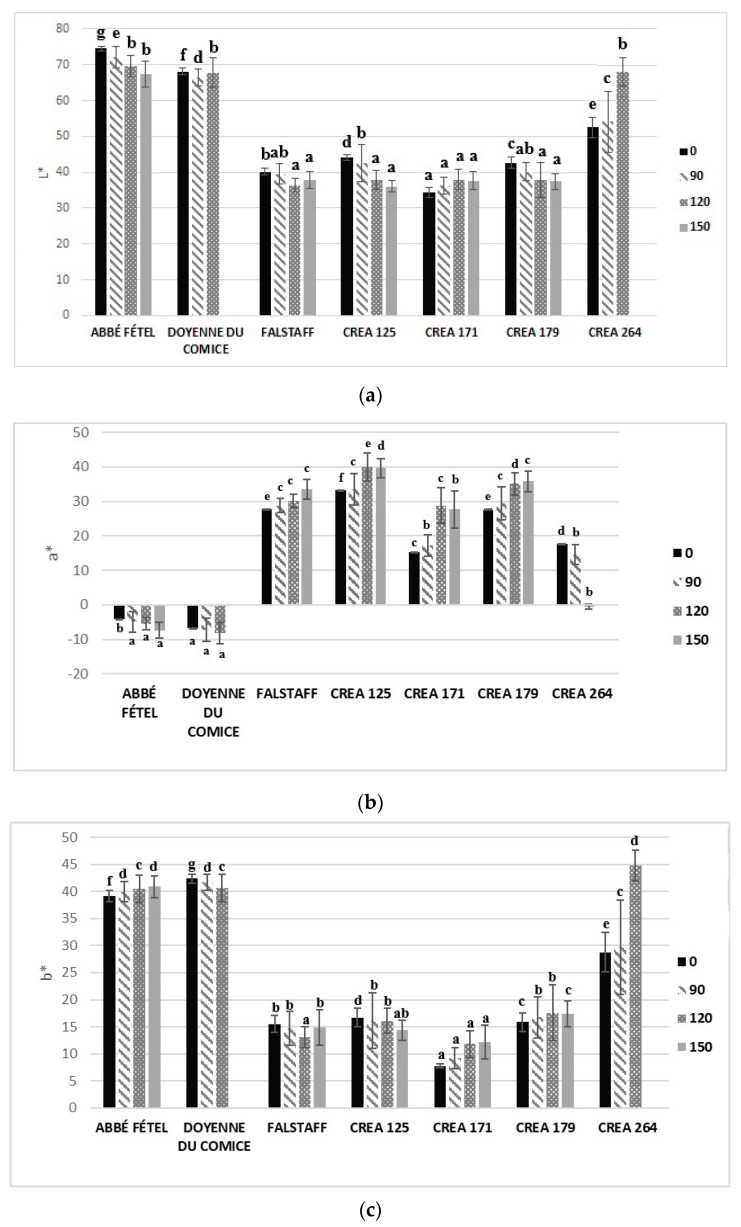

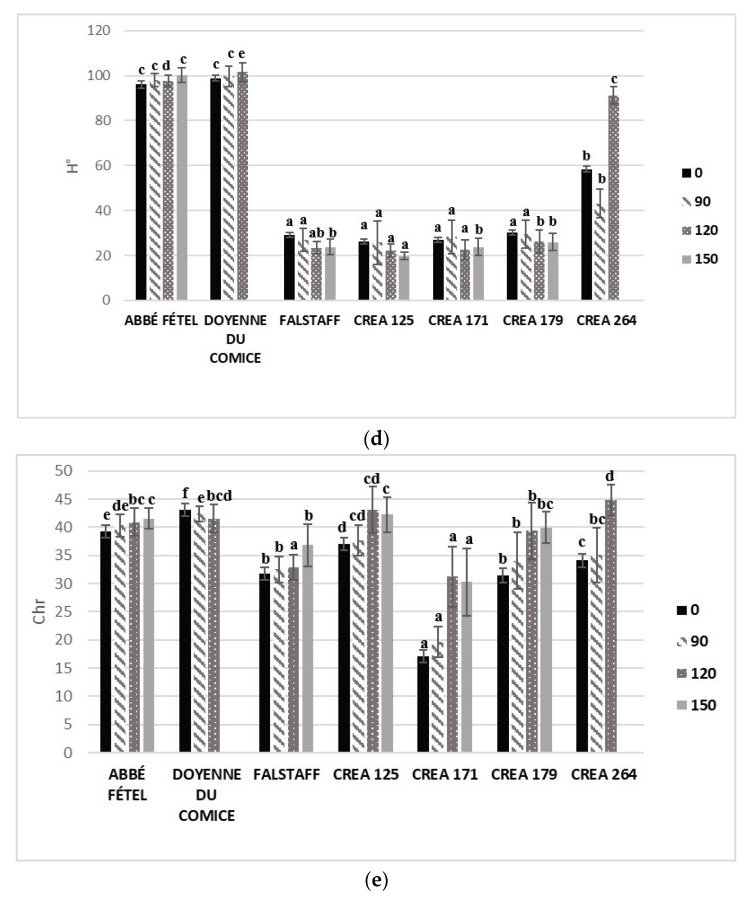
Color parameters L* (**a**), a* (**b**), b* (**c**), hue angle (**d**), chroma (**e**), in different pear cultivars/selections at harvest (0 days), and after 90, 120, and 150 days of storage at −1 °C ± 0.5 °C. Statistical comparisons were made using data from 90, 120, and 150 days. Means followed by the same letter do not differ significantly at *p* = 0.05 (Duncan test). Statistical comparisons were made within each timing.

‘Falstaff’, ‘CREA 125’, ‘CREA 171’, and ‘CREA 179’ are red peel cultivars/selections, and the red color is spread over 75% of the peel; ‘CREA 264’ is red blushed pear with 40% of red color on the peel surface, while ‘Abbé Fétel’ and ‘Doyenne du Comice’ have a green peel at harvest, that then becomes green-yellow or yellow when ripe. L* represents the relative lightness of color with a range from 0 to 100, with small values indicating dark colors and large values for bright. a* value is negative for fruits with green peel, like in Abbé Fétel and ‘Doyenne du Comice’, and positive for red color peel, like in ‘Falstaff’, ‘CREA 125’, ‘CREA 171’, ‘CREA 179’, and ‘CREA 264’. There were statistical differences in color vividness, chroma index, between different cultivars/selections. ‘CREA 171’ had the lowest values at harvest and during the whole CS period, only increasing over time. Highlighted less dark color confirmed also by L* value, that were lower in all red peel varieties and selections. Color changes from green to yellow were indicated by calculating the hue angle (H°), red peel cultivars/selections had the lowest H° angles at harvest and during the entire CS period (Figure 1).

### 3.2. Nutraceutical Traits in Different Cultivar/Selection Pears During Cold Storage

Pear fruits contain, in different amounts based on the cultivar considered, numerous nutrients such as vitamin C, vitamin E, β-carotene, polyphenols, sugars, organic acids, amino acids, and fatty acids [27].

Several studies have demonstrated that polyphenols play an important role in antioxidant activity [43]. This class of compounds is responsible for antioxidative, anti-inflammatory, and anticancer activities, and plays a protective role against cardiovascular diseases and cataracts and are properties which are attributed to functional fruits [43]. Polyphenol content depends on many factors, such as cultivar, stage of maturity, storage conditions, and post-harvest treatments [44].

**Figure 2:**
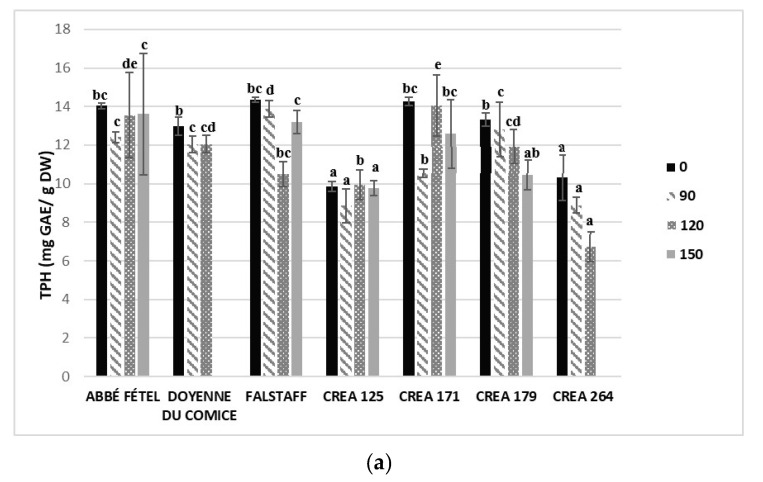

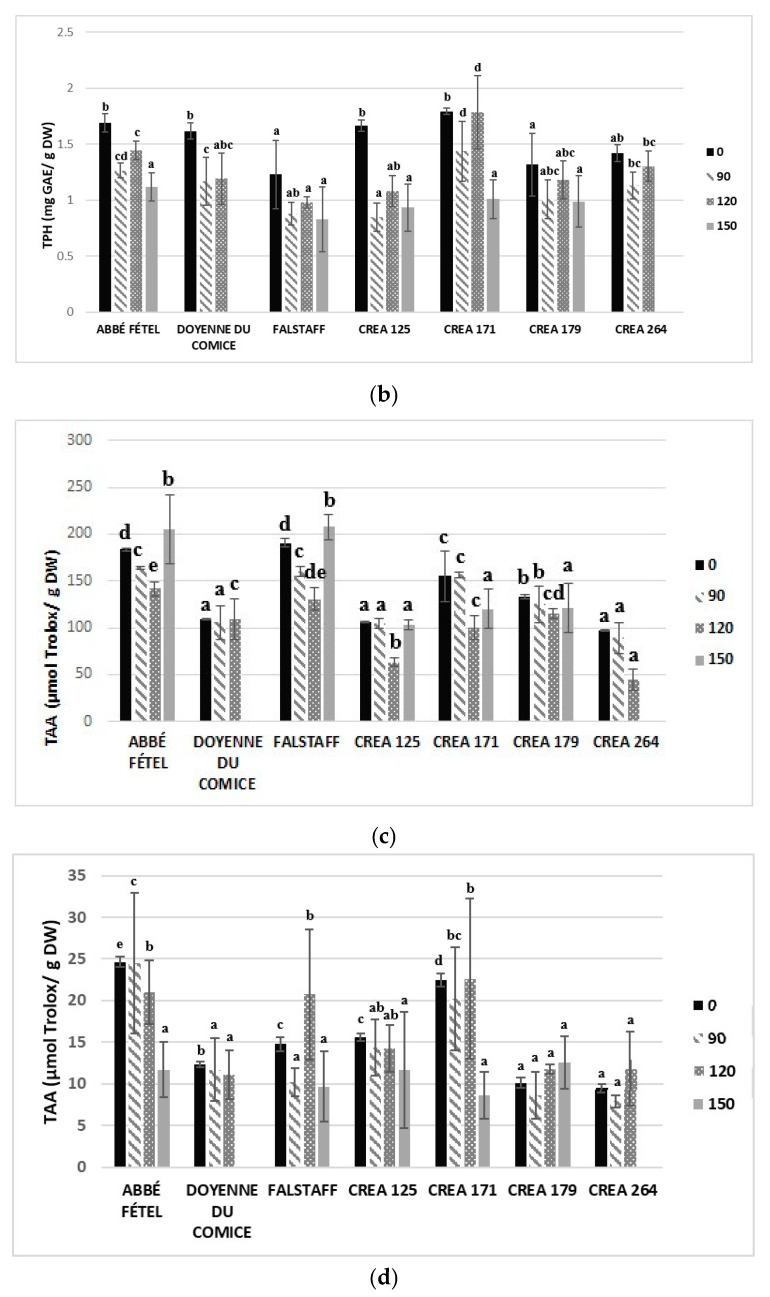
Total polyphenol content (µmol GAE/g DW) in peel (**a**) and in flesh (**b**), and total antioxidant activity (µmol Trolox/ g DW) in peel (**c**) and in flesh (**d**), in different cultivars/selections of pears at harvest (0 days) and after 90, 120 and 150 days of storage at −1 °C ± 0.5 °C. Statistical comparisons were made using data from harvest (0 day), and after 90, 120, and 150 days. Means followed by the same letter do not differ significantly at *p* = 0.05 (Duncan test). Statistical comparisons were made within each timing.

At harvest, ‘Abbé Fétel’ (14.04 ± 0.16 mg GAE/g DW), ‘Falstaff’ (14.37 ± 0.13 mg GAE/g DW) and ‘CREA 171’ (14.27 ± 0.22 mg GAE/g DW) showed the highest TPH content in the peel, followed by ‘Doyenne du Comice’ (12.97 ± 0.47 mg GAE/g DW) and ‘CREA 179’(13.34 ± 0.34 mg GAE/g DW), with a weak statistical difference. The lowest TPH in peel was detected in ‘CREA 264’ (10.31 ± 1.19 mg GAE/g DW) and in ‘CREA 125’ (9.87 ± 0.25 mg GAE/g DW). The TPH content in flesh was lower than in peel and ‘Abbé Fétel’ (1.69 ± 0.08 mg GAE/g DW), ‘Doyenne du Comice’ (1.62 ± 0.07 mg GAE/g DW), ‘CREA 125’ (1.66 ± 0.05 mg GAE/g DW) and ‘CREA 171’ (1.79 ± 0.03 mg GAE/g DW) reached the highest values. To attain a more complete understanding of antioxidants to reduce superficial scald development in three-storage timing (90,120 and 150 days) of the seven pear cultivars and selections, the TAA and TPH content in peel and pulp (Figure 2). TPH content was evaluated in both peel and flesh of the tested pear cultivars/selections. After 90 days of CS, ‘Falstaff’ (13.6 ± 1.6 mg GAE/g D.W.) and ‘CREA 179’ (12.8±1.4 mg GAE/g D.W.) showed the highest TPH in peel, followed by ‘Doyenne du Comice’ and ‘Abbé Fétel’, and the lowest TPH was detected in ‘CREA 264’ (8.6 ± 1.4 mg GAE/g D.W.). After 120 and 150 days, ‘Abbé Fétel ‘and ‘CREA 171’ showed the highest TPH in peel. ‘CREA 264’ and ‘CREA 125’ showed lowest TPH during the three CS timings (Figure 2a). The highest content of TPH in fruit flesh was always statistically different (*p* < 0.05). In ‘Abbé Fétel’ and ‘CREA 171’ instead in ‘Falstaff’ and in ‘CREA 179’ were observed the lowest ones (Figure 2b).

Several classes of phenolic compounds in pear cultivars were identified [45] and several studies have demonstrated that these compounds are associated with resistance to scald development in apples and pears [11,46]. Kolniak-Ostek [47] highlighted that the polyphenols, mainly present in ‘Radana’ pears, were caffeic acid and its derivatives (27.0 ± 0.1 and 65.1 ± 0.2 mg/100 g DW), monomeric catechins (31.7 ± 0.1 and 92.5 ± 0.2 mg/100 g DW), polymeric procyanidins (138.1 ± 0.2 and 352.8 ± 0.5 mg/100 g DW), and arbutin (31.7 ± 0.1 and 92.5 ± 0.2 mg/100 g DW), in pulp and peel, respectively.

Regarding TAA, at harvest ‘Falstaff’ (190.61 ± 4.52 µmol TROLOX/g DW) and ‘Abbé Fétel’ (183.32 ± 0.84 µmol TROLOX/g DW) showed the highest TAA in peel and also after 90 days of CS, ‘Falstaff’ (160.08± 4.95 µmol TROLOX/g D.W.) and ‘Abbé Fétel’ (164±1.3 µmol TROLOX/g D.W.) showed the highest TAA in peel, followed by ‘CREA 171’, and the lowest value of TAA was detected in ‘CREA 264’. After 120 and 150 days, ‘Abbé Fétel’ and ‘Falstaff’ showed the highest TAA in peel. ‘CREA 264’ and ‘CREA 125’ showed the lowest value of TAA at harvest and across three CS timings (Figure 2c). The highest flesh TAA content was detected in ‘Abbé Fétel’ and ‘CREA 171’ at harvest and after 90 days of CS; however, after 150 days, the TAA was uniform among all cultivar/selections without significant difference (*p* < 0.05). The lowest flesh TAA was observed in ‘CREA 179’ and ‘CREA 264’ at harvest and during the CS period (Figure 2d).

Fruit sensitivity to superficial scald is determined by endogenous antioxidant potential [2]. TAA values were correlated with TPH, being mainly associated with antioxidant capacity in pear fruits.

### 3.3. Antioxidantenzymes in Flesh and Peel of Different Cultivar/Selection Pears During Cold Storage

To investigate the relationship between superficial scald incidence in several pear cultivars/selections and antioxidant systems, the activity of three key antioxidant enzymes SOD, CAT, and APX was assessed in peel and pulp samples.

**Figure 3:**
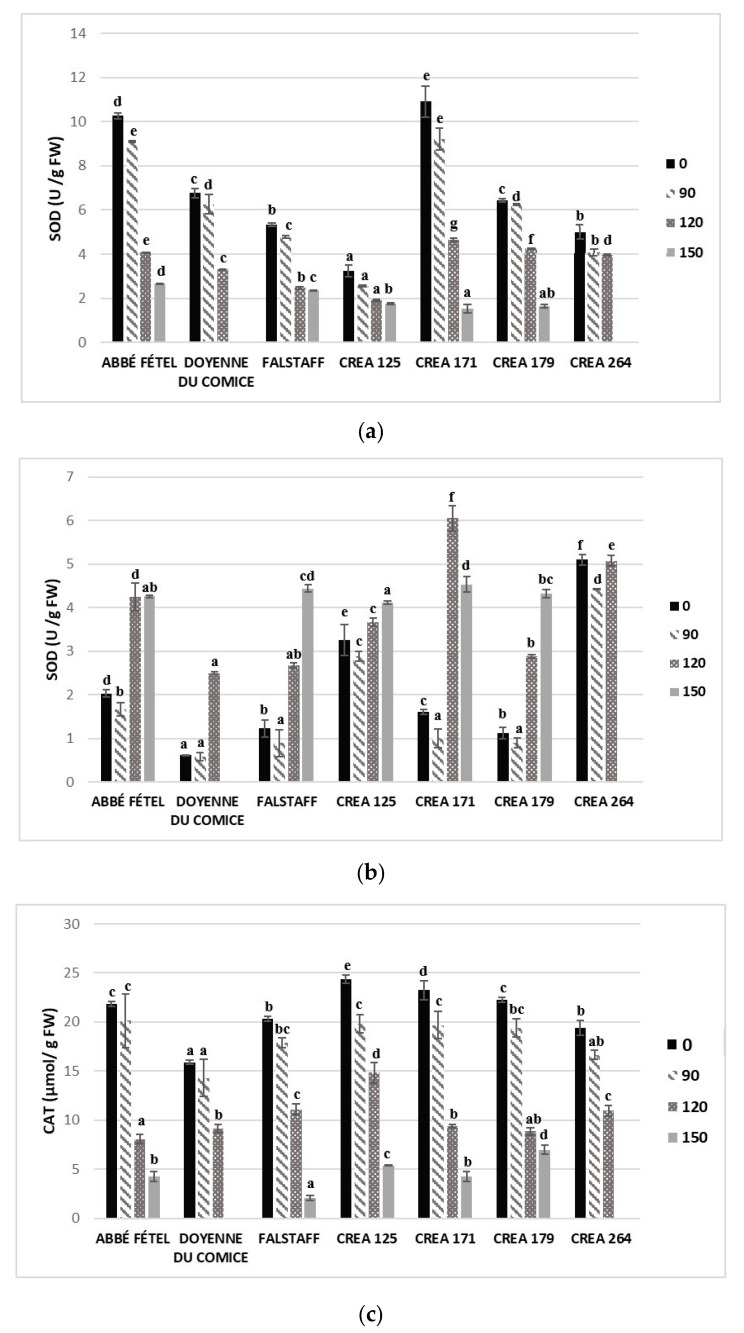

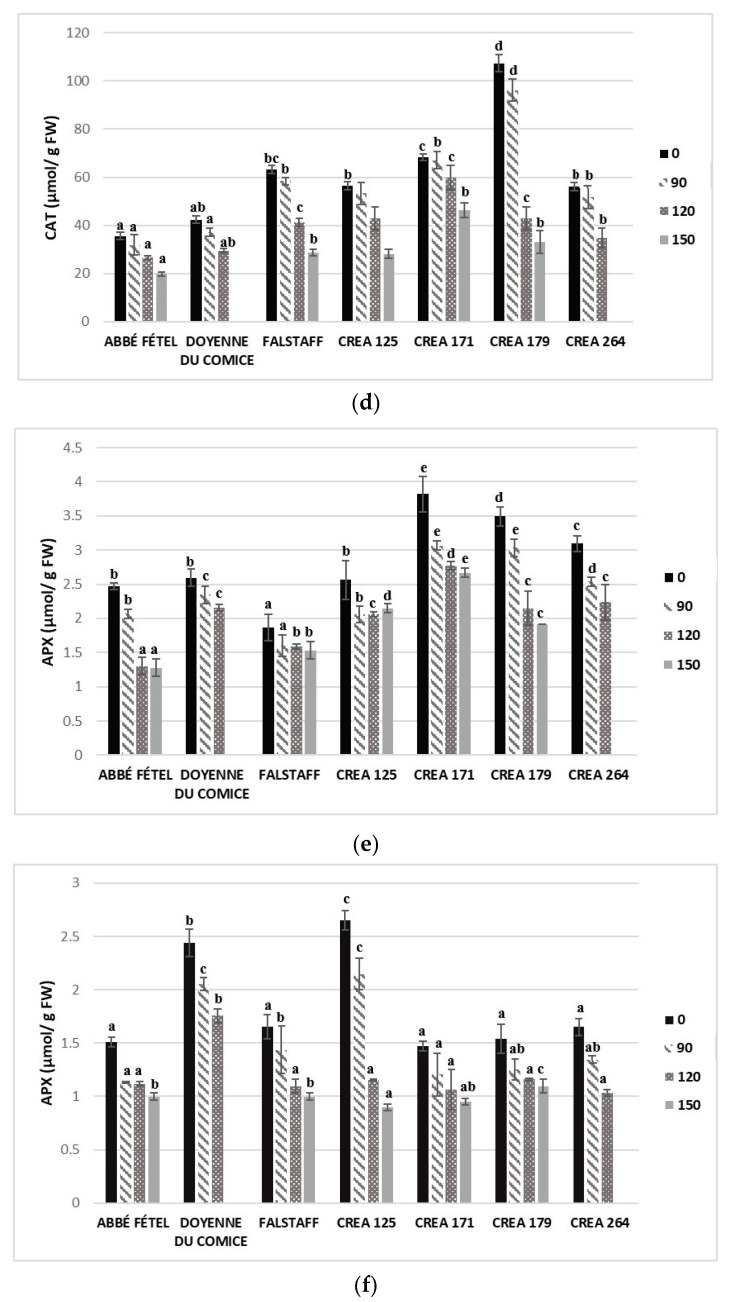
Superoxide dismutase (U/g FW) in peel (**a**) and flesh (**b**), Catalase (µmol/ g FW)in peel (**c**) and flesh (**d**) and ascorbate peroxidase(µmol/g FW) in peel (**e**) and flesh (**f**) in different cultivar/selection of pears at harvest (0 days), and after 90, 120 and 150 days of storage at −1 °C ± 0.5 °C. Means followed by the same letter do not differ significantly at *p* = 0.05 (Duncan test). Statistical comparisons were made within each timing.

SOD is the first ROS scavenging enzyme involved in the dismutation of toxic oxygen (radical superoxide) to H_2_O_2_ and molecular oxygen (O_2_) [48]. Significant difference in SOD activity was detected among pear cultivars/selection at harvest (Figure 3a,b). In peel, ‘CREA125’ showed the lower value while the higher one was registered in ‘CREA171’.

SOD activity in the peel of multiple pear cultivars/selections decreased throughout CS. After three months of CS, SOD activity showed significant differences among samples and the lowest mean value of 2.56 ± 0.04 U g^−1^ FW was observed in ‘CREA 125’, compared to that in other cultivars/selections, indicating a low ROS production in this pear selection (Figure 3a). SOD activity in pear flesh showed significant differences (*p* < 0.05) at harvest. After 90 days of CS, this enzyme displayed the lower and higher values in ‘Doyenne du Comice’ (0.58 ± 0.09 U g^−1^ FW) and ‘CREA 264’ (4.42 ± 0.06 U g^−1^ FW), respectively. SOD activity increased in ‘CREA 171’ and ‘CREA 264’ after 120 days of CS, while the other samples registered lower SOD activity at 120 days compared to that after 150 days of CS (Figure 3b). As suggested by Sarkar et al. [15], high SOD activity in apples could be due to a high ROS production and this high amount of ROS may have triggered a high SOD response. Furthermore, pre-harvest conditions such as fruit sunlight-exposure improved SOD activity, thus reducing superficial scald development in ‘d’Anjou’ pears during five months of CS [11]. 1-MCP post-harvest treatment alone or combined with MAP condition allowed pears to maintain higher levels of SOD activity, therefore reducing scald incidences after day 180 and 120 of CS in Yali and ‘Wujiuxiang’ pears, respectively [11,18]. Low levels of SOD activity contributed to major post-harvest disorder development in pear cultivars [49].

H_2_O_2_ is further converted to harmless water by APX and CAT, which belong to two different classes of H_2_O_2_-scavenging enzymes with different affinities to the substrate [48]. APX reduces H_2_O_2_ to H_2_O by utilizing ascorbate as an electron donor, and ascorbate is then regenerated by the ascorbate-glutathione cycle [34]. CAT activity showed the highest values at harvest and decreased in the peel and flesh of different pear cultivars/selections during storage, with higher values in flesh compared to that in peel. The highest CAT activity change rates of 55.4% and 59.8% were registered in ‘CREA 179’ pear flesh and in ‘Abbé Fétel’ pear peel between 90 and 120 days of CS, respectively (Figure 3c,d). ‘CREA 179’ pear peel and ‘Abbé Fétel’ pear flesh also showed the lowest CAT activity change rates of 21.0% and 25.4% between 120 and 150 days of CS, respectively (Figure 3c,d).

Higher CAT activity was registered in scald-resistant than that in scald-susceptible apple cultivars [50], and in pear fruits with a low scald incidence [11].

APX activity showed the highest values at harvest and decreased throughout CS in all pear cultivars/selections in both peel and flesh. The highest values were registered in ‘CREA 171’ (3.07 ± 0.06 µmolg^−1^ FW) and ‘CREA 179’ peel (3.03 ± 0.13 µmolg^−1^ FW), and in ‘CREA 125’ (2.15 ± 0.15 µmolg^−1^ FW) and ‘Doyenne du Comice’ (2.05 ± 0.06 µmolg^−1^ FW) flesh, after 90 days of CS, while the lowest values were observed in ‘Abbé Fétel’ (1.28 ± 0.13 µmolg^−1^ FW) and ‘CREA 125’ (0.90 ± 0.03 µmolg^−1^ FW) peel after 150 days of CS (Figure 3e,f).

Ascorbate is the substrate of APX, and its availability is essential for enzyme activity under cold stress. Furthermore, ascorbate plays a key role in explaining the differences in scald susceptibility between pear cultivars [7]. APX activity was higher in scald-resistant pears than that in scald-susceptible ones, with different sunlight-exposure [11], and in ‘Wujiuxiang’ pears treated with 1-MCP with low scald incidence [18].

The ROS processing system and its related gene network during post-harvest cold acclimation promotes fruit resistance to oxidative stress and limits superficial scald development in apples [51].

### 3.4. Oxidative Damage in Flesh and Peel of Different Cultivar/Selection Pears During Cold Storage

Cold stress induces excessive accumulation of ROS that is responsible for the changes in membrane lipid composition due to their oxidation [52]. During CS the decrease of antioxidant enzymes such as SOD, CAT, and APX causes oxidative damage altering the stability of the cell membrane with a consequent increase in LOX activity and enzymatic browning due to GPX and PPO activity.

**Figure 4:**
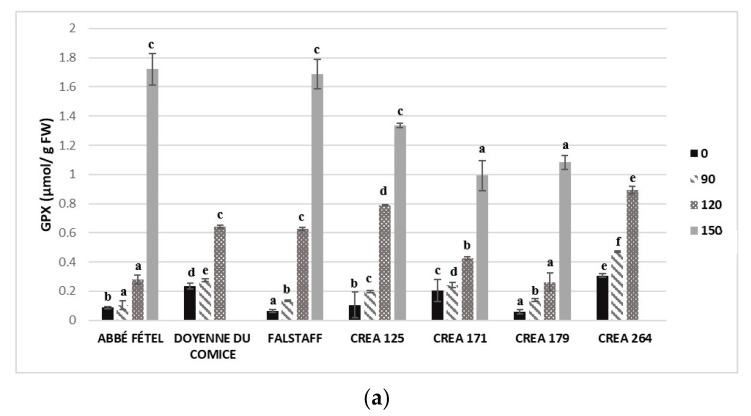

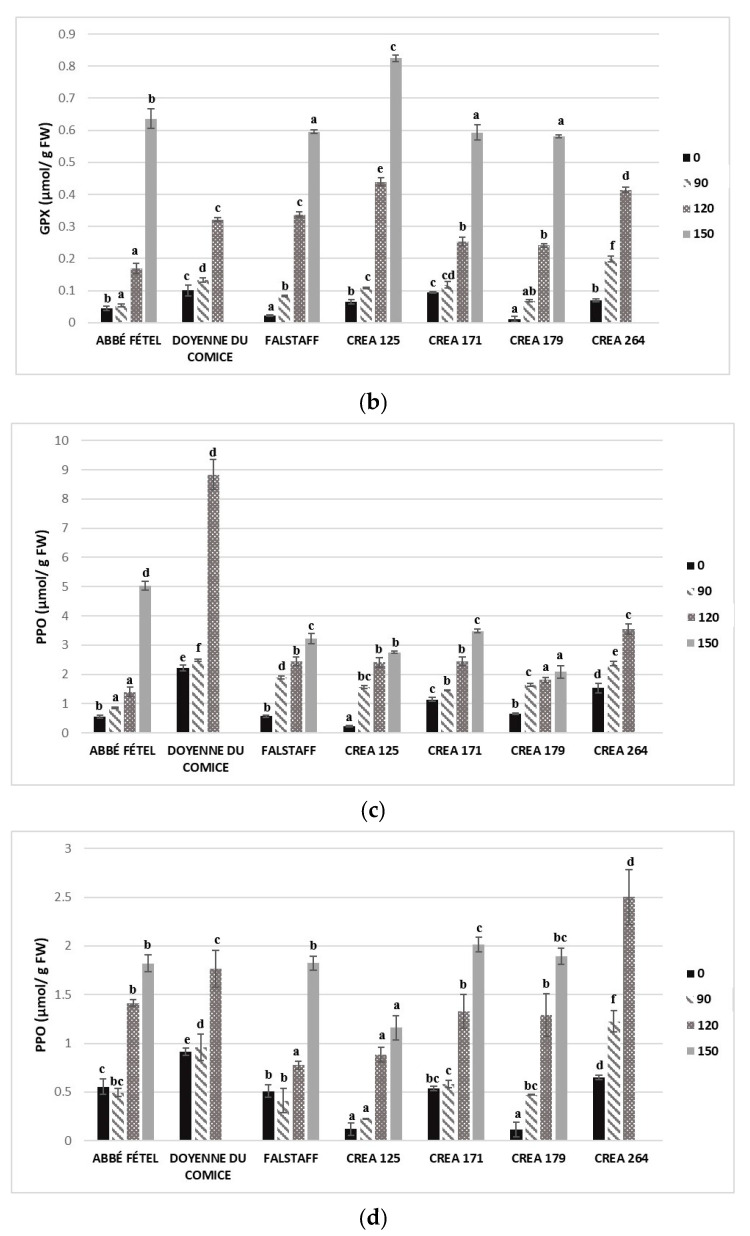

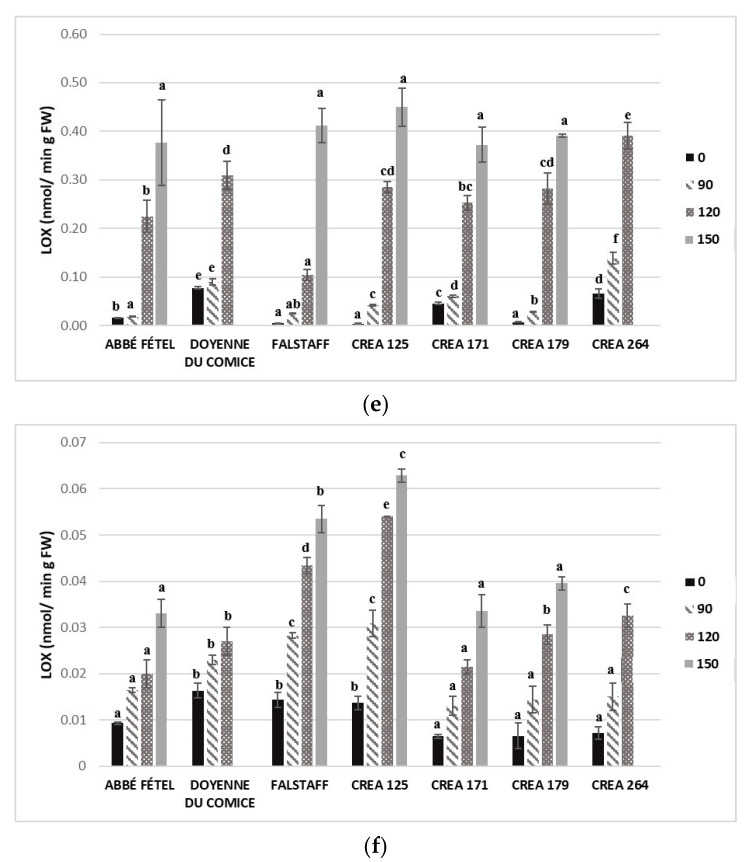
Guaiacol peroxidase(µmol/ g FW) in peel (**a**) and flesh (**b**), polyphenol oxidase (µmol/ g FW) in peel (**c**) and flesh (**d**) and lipoxygenase (nmol/min g FW) in peel (**e**) and flesh (**f**) in different cultivar/selection of pears at harvest (0 day), and after 90, 120 and 150 days of storage at −1 °C ± 0.5 °C. Means followed by the same letter do not differ significantly at *p* = 0.05 (Duncan test). Statistical comparisons were made within each timing.

GPX activity, in peel and flesh of fruit, showed higher values in scald-susceptible pear cultivars/selections. Significant differences in GPX activity were registered among pear cultivars/selection at harvest and the increase of enzyme activity occurred during CS (Figure 4a,b).

PPO was significantly different in the flesh and peel of pear samples with higher values in peel compared to that in flesh. Pear cultivar/selections showed an increase in PPO activity during CS, the lowest value was detected in ’Abbé Fétel’ peel (0.85 ± 0.03 µmolg^−1^ FW) after 90 days, while the highest value was detected in ‘Doyenne du Comice’ (8.84 ± 0.52 µmolg^−1^ FW) after 120 days (Figure 4c,d).

LOX was closely correlated with lipid peroxidation during fruit ripening, and its activity was involved in scald development. LOX activity showed lower values at harvest in pear cultivars/selections and its activity increased during CS with higher values in peel (about 10-fold) compared to that in flesh. This suggests that membrane damage resulting from lipid peroxidation was greater in pear peel compared to that in pear flesh. The highest values of LOX activity were registered in scald-susceptible pears such as ‘Doyenne du Comice’ (0.31 ± 0.03 nnolg^−1^ FW) and ‘CREA 264’ (0.40 ± 0.03 nnolg^−1^ FW) after 120 days of CS (Figure 4e,f).

GPX is another important antioxidant enzyme belonging to the peroxidases, which catalyzes single-electron oxidation of several antioxidant compounds with hydrogen peroxide, and it is also involved in superficial scald susceptibility as demonstrated in apples [50]. PPO and LOX are involved in scald development as suggested by several studies in which ‘Empire’ apples and ‘Dangshansuli’ pears were treated with diphenylamine (DPA) as post-harvest treatment to control this disorder. DPA has a double effect on the metabolization of α-farnesene and conjugated trienes, and on the inhibition of LOX and PPO activities [53,54]. Higher GPX and LOX activity were detected in Japanese [55] and ‘Flord’Hivern’ pears [6], suggesting an enhanced lipid peroxidation and hence partially explaining the scald development.

### 3.5. PCA to Evaluate the Difference in Different Cultivar/Selection Pears During Cold Storage

**Figure 5:**
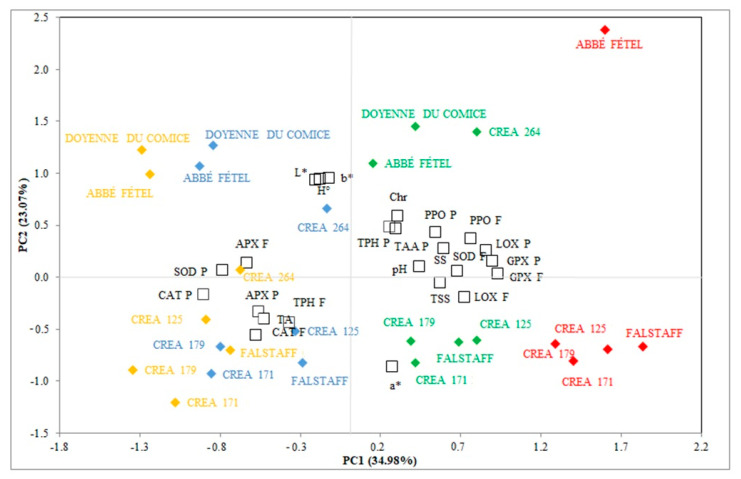
Principal component analysis of the physico-chemical, nutraceutical, and enzymatic traits in peel (P) and flesh (F) of seven cultivar/selection pears at harvest (♦), and after 90 (♦), 120 (♦) and 150 (♦) days of storage at −1 °C ± 0.5 °C (L*: lightness; H°: hue angle; Chr: chroma; TA: titratable acidity; TSS: total soluble solids; SS: superficial scald; TAA: total antioxidant activity; TPH: total phenolic content; SOD: superoxide dismutase; CAT: catalase; APX: ascorbate peroxidase; GPX: guaiacol peroxidase; PPO: polyphenol oxidase and LOX: lipoxygenase).

PCA was applied to define principal components (PCs) to better describe the overall variance in all analyzed parameters. The first two principal components explained 58.05% of the variation (34.98% and 23.07% for PC1 and PC2, respectively) (Figure 5). GPX P (*r^2^* = 0.901) and F (*r^2^* =0.937), PPO P (*r^2^* = 0.547) and F (*r^2^* = 0.767), LOX P (*r^2^* = 0.861) and F (*r^2^* = 0.728), SOD F (*r^2^* = 0.683), TSS (*r^2^* = 0.573), SS (*r^2^* = 0.599) and pH (*r^2^* = 0.445) showed a positive correlation with PC1; while CAT P (*r^2^* = −0.898) and F (*r^2^* = −0.573), SOD P (*r^2^* = −0.779), APX P (*r^2^* = −0.559), TA (*r^2^* = −0.524), TAA F (*r^2^* = −0.444) and TPH F (*r^2^* = −0.440) were negatively correlated with PC1. b* (*r^2^* = 0.948), L* (*r^2^* = 0.930), Chr (*r^2^* = 0.587), H° (*r^2^* = 0.940) and TAA P (*r^2^* = 0.466) and TPH P (*r^2^* = 0.481) were positively correlated with PC2; while a* (*r^2^* = −0.869) showed a negative correlation with PC2.

Pear cultivars/selections were grouped differently in 2D-PCA plot (Figure 5). At harvest, Abbé Fétel’, ‘Doyenne du Comice’ and ‘CREA 264’ were plotted in the PC1 negative and PC2 positive dial, while ‘Falstaff’, ‘CREA 125’, ‘CREA 179’ and ‘CREA 171’ were projected on the dial defined by PCs negative. The PC scores at 90 days of storage resulted place between II and III dials shifted towards negative values of PC1, whereas the samples stored for 120 days are placed between the four quadrants. Towards positive values of PC1, the scores of the different cultivars/selections of pear stored for 150 days were found between I and IV dials. Furthermore, pear cultivars/selections were separated based on similarities in the analyzed traits.

This multivariate analysis allowed us to highlight the physico-chemical, nutraceutical, and enzymatic changes in seven pear cultivars/selections with different superficial scald susceptibilities, during CS. PCA is a valid tool that has been applied in other studies to evaluate the effectiveness of post-harvest treatments on qualitative decay and oxidative damages in pomegranates, sweet cherries, figs, and loquats [14,37,56,57].

## 4. Conclusions

Seven different pear cultivars/selections analyzed in this study showed physico-chemical and qualitative changes during three months of CS, and different superficial scald susceptibilities. After 150 d of CS, ‘Doyenne du Comice’ and ‘CREA 264’ showed the highest superficial scald susceptibility, while ‘Abbé Fétel’, ‘CREA 179’, ‘CREA 171’, and ‘Falstaff’ showed high resistance to superficial scald susceptibility. Furthermore, antioxidant enzymes and other bioactive compounds have an important role to play in the occurrence of superficial scald susceptibility/resistance. The antioxidant system alleviates oxidative damage and, in addition to other biochemical features, could be involved in determining pear susceptibility/resistance to superficial scald development. During CS, the decrease of antioxidant enzymes, such as SOD, CAT, and APX, causes oxidative damage, thus altering the stability of the cell membrane with a consequent increase of LOX activity, and increasing the enzymatic browning due to GPX and PPO activity. GPX activity, in both the peel and flesh of fruit, showed higher values in scald-susceptible pear cultivars/selections. The highest GPX values were detected in ‘CREA 264’ and ‘Doyenne du Comice’ after 90 days of CS. ‘Doyenne du Comice’ showed the highest PPO value after 120 days of CS. LOX activity of pear cultivars/selections increased during CS with higher values in peel (about 10-fold) compared to that in flesh. This suggests that membrane damage resulting from lipid peroxidation was greater in pear peel compared to that in pear flesh. The highest values of LOX activity were registered in scald-susceptible pears such as ‘Doyenne du Comice’ and ‘CREA 125’ after 120 days of CS.

Understanding different scald susceptibilities of pear cultivars/selections may enable us to improve post-harvest treatments that could be used to reduce scald in this fruit crop.

## Figures and Tables

**Table 1:** Harvest time and fruit characteristics of the analyzed pear cultivars and advanced selections.

Pear Cultivar/Advanced Selection	Harvest Date	Firmness at Harvest (kg 0.5 cm^−2^)	Shape	Color and Overcolor, %	Russeting, %	Maximum Diameter (mm)
ABBÉ FÉTEL	22 August 2018	5.5	Oblong pyriform	Green–yellow	10	72
DOYENNE DU COMICE	26 August 2018	5.5	Short elongated	Green–yellow	-	75
FALSTAFF	24 August 2018	5.5	Oblong pyriform	Green–yellow, 75% red	-	75
CREA 125	19 August 2018	5.3	Quince shaped	Green–yellow, 85% red	-	68
CREA 171	15 September 2018	6.0	Oblong ovate Pyriform	Dark red	50	75
CREA 179	25 August 2018	5.0	Short elongated	Green–yellow, 90% red	-	78
CREA 264	13 August 2018	5.2	Oblong ovate pyriform	Green–yellow, 40% red blushed	-	75

**Table 2:** Physico-chemical traits of different cultivar/selection pears at harvest (0 day), and after 90, 120 and 150 days of storage at −1 °C ± 0.5 °C.

Cultivar/Selection	Storage Time (days)	TSS (°Brix)	TA (g Malic Acid/L)	pH	SS (%)
ABBÉ FÉTEL	0	14.43 ± 0.12 (e)	1.84 ± 0.09 (a)	4.51 ± 0.11 (cd)	0 (a)
DOYENNE DU COMICE		11.55 ± 0.10 (a)	2.59 ± 0.05 (c)	4.05 ± 0.12 (b)	0 (a)
FALSTAFF		13.31 ± 0.25 (c)	2.19 ± 0.18 (b)	4.61 ± 0.01 (d)	0 (a)
CREA 125		12.58 ± 0.26 (b)	2.55 ± 0.11 (c)	4.45 ± 0.06 (c)	0 (a)
CREA 171		15.50 ± 0.15 (f)	3.91 ± 0.05 (e)	3.78 ± 0.10 (a)	0 (a)
CREA 179		12.60 ± 0.22 (b)	3.28 ± 0.23 (d)	4.11 ± 0.04 (b)	0 (a)
CREA 264		13.77 ± 0.07 (d)	2.64 ± 0.06 (c)	4.38 ± 0.02 (c)	0 (a)
ABBÉ FÉTEL	90	15.62 ± 0.91 (cd)	1.58 ± 0.44 (a)	4.72 ± 0.17 (e)	0 (a)
DOYENNE DU COMICE		12.93 ± 1.45 (a)	2.25 ± 0.19 (bc)	4.13 ± 0.15 (ab)	55.00 ± 10.00 (d)
FALSTAFF		14.97 ± 0.39 (bc)	1.91 ± 0.29 (ab)	4.76 ± 0.06 (e)	0 (a)
CREA 125		13.07 ± 0.43 (a)	2.30 ± 0.13 (bc)	4.52 ± 0.11 (d)	15.00 ± 5.00 (b)
CREA 171		16.56 ± 0.77 (d)	3.65 ± 0.48 (e)	3.99 ± 0.17 (a)	0 (a)
CREA 179		13.98 ± 0.56 (ab)	3.11 ± 0.19 (d)	4.24 ± 0.03 (bc)	45.00 ± 5.00 (c)
CREA 264		14.90 ± 0.56 (bc)	2.45 ± 0.29 (c)	4.32 ± 0.05 (c)	73.00 ± 3.00 (e)
ABBÉ FÉTEL	120	15.70 ± 0.62 (b)	1.37 ± 0.25 (a)	4.74 ± 0.01 (de)	33.00 ± 7.55 (a)
DOYENNE DU COMICE		14.92 ± 0.58 (b)	1.82 ± 0.53 (ab)	4.67 ± 0.09 (d)	66.00 ± 6.00 (b)
FALSTAFF		15.75 ± 0.62 (b)	1.70 ± 0.28 (ab)	4.85 ± 0.12 (e)	33.00 ± 5.00 (a)
CREA 125		13.46 ± 0.40 (a)	2.01 ± 0.21 (b)	4.47 ± 0.07 (c)	33.00 ± 7.55 (a)
CREA 171		17.15 ± 1.07 (c)	3.25 ± 0.63 (c)	4.03 ± 0.06 (a)	33.30 ± 2.51 (a)
CREA 179		14.72 ± 0.43 (b)	3.03 ± 0.17 (c)	4.24 ± 0.10 (b)	60.00 ± 5.00 (b)
CREA 264		15.10 ± 0.45 (b)	2.00 ± 0.45 (b)	4.50 ± 0.12 (c)	80.00 ± 2.00 (c)
ABBÉ FÉTEL	150	17.15 ± 1.07 (b)	1.64 ± 0.19 (b)	4.32 ± 0.09 (a)	36.67 ± 3.51 (ab)
DOYENNE DU COMICE		-	-	-	100 (d)
FALSTAFF		17.07 ± 1.65 (b)	1.37 ± 0.12 (a)	4.93 ± 0.04 (d)	40.00 ± 2.00 (b)
CREA 125		13.90 ± 0.20 (a)	1.82 ± 0.10 (b)	4.57 ± 0.05 (b)	36.00 ± 1.73 (ab)
CREA 171		17.77 ± 0.73 (b)	1.15 ± 0.15 (a)	4.79 ± 0.08 (c)	35.00 ± 2.00 (a)
CREA 179		14.90 ± 0.51 (a)	2.55 ± 0.13 (c)	4.28 ± 0.04 (a)	67.30 ± 2.52 (c)
CREA 264		-	-	-	100 (d)

TSS: total soluble solids; TA: titratable acidity; pH; SS: superficial scald. Statistical comparisons were made within each timing. Means followed by the same letter do not differ significantly at *p* = 0.05 (Duncan test).
